# Differences in time to task failure and fatigability between children and young adults: A systematic review and meta-analysis

**DOI:** 10.3389/fphys.2022.1026012

**Published:** 2022-10-31

**Authors:** Robin Souron, Marion Carayol, Vincent Martin, Enzo Piponnier, Pascale Duché, Mathieu Gruet

**Affiliations:** ^1^ Université de Toulon, Laboratoire IAPS (n°201723207F), Toulon, France; ^2^ Nantes Université, Movement—Interactions—Performance, MIP, UR 4334, Nantes, France; ^3^ Université Clermont-Auvergne, Laboratoire AME2P (EA 3533), Clermont-Ferrand, France; ^4^ Institut Universitaire de France, Paris, France; ^5^ Université Côte d’Azur, LAMHESS (EA 6312), Nice, France

**Keywords:** children, adolescents, fatigability, time to task failure, neuromuscular physiology

## Abstract

The transition from childhood to adulthood is characterized by many physiological processes impacting exercise performance. Performance fatigability and time to task failure are commonly used to capture exercise performance. This review aimed to determine the differences in fatigability and TTF between youth (including both children and adolescents) and young adults, and to evaluate the influence of exercise modalities (i.e., exercise duration and type of exercise) on these differences. Medline, SPORTDiscus and Cochrane Library were searched. Thirty-four studies were included. The meta-analyses revealed that both children (SMD −1.15; *p* < 0.001) and adolescents (SMD −1.26; *p* = 0.022) were less fatigable than adults. Additional analysis revealed that children were less fatigable during dynamic exercises (SMD −1.58; *p* < 0.001) with no differences during isometric ones (SMD –0.46; *p* = 0.22). Children (SMD 0.89; *p* = 0.018) but not adolescents (SMD 0.75; *p* = 0.090) had longer TTF than adults. Additional analyses revealed 1) that children had longer TTF for isometric (SMD 1.25; *p* < 0.001) but not dynamic exercises (SMD −0.27; *p* = 0.83), and 2) that TTF differences between children and adults were larger for short- (SMD 1.46; *p* = 0.028) than long-duration exercises (SMD 0.20; *p* = 0.64). Children have higher endurance and are less fatigable than adults. These differences are influenced by the exercise modality, suggesting distinct physiological functioning during exercise between children and adults. The low number of studies comparing these outcomes between adolescents versus children and adults prevents robust conclusions and warrants further investigations in adolescent individuals.

## 1 Introduction

There has been a growing interest in recent decades for the evaluation of exercise-induced fatigue in children, with numerous reports investigating potential child-adult differences regarding its magnitude and etiology (Ratel et al., 2006a; Patikas et al., 2018).

Fatigue is a multifactorial and complex concept, and a new taxonomy has been recently proposed to acknowledge its attributes that are performance and perceived fatigability (this latter also referred to as perception of fatigue). Those two attributes are closely interrelated and inseparable (Kluger et al., 2013; Enoka and Duchateau, 2016; Gruet, 2018). Perceived fatigability/perception of fatigue can refer to a feeling of reduced capacity to cope with physical or mental stressors (Micklewright et al., 2017) and is related to the maintenance of homeostasis and the psychological state of the individual (Kluger et al., 2013; Enoka and Duchateau, 2016). Performance fatigability refers to a decline in an objective measure of performance (e.g., muscle force or power) during and/or after a given exercise, hereafter referred to as fatigability. Although linked but not interchangeable, fatigability should not be confused with another commonly-used term when exercise-induced fatigue is investigated, i.e., time to task failure (TTF). This term refers to the capacity for a subject to perform an exercise at a given percentage of a maximal parameter (e.g., muscle force, maximal aerobic power) over an extended period of time until failure.

Over the last three decades, several studies investigated differences in TTF [e.g., Berthoin et al. (2003); Barker et al. (2010); Leclair et al. (2011); Patikas et al. (2013); Hatzikotoulas et al. (2014); Tanina et al. (2017); Piponnier et al. (2018); Bar-Yoseph et al. (2019)] and fatigability [Pullinen et al. (2011); Patikas et al. (2013); Hatzikotoulas et al. (2014); Murphy et al. (2014); Willcocks et al. (2014); Lazaridis et al. (2018)] between youth (that includes both children and adolescents) and adults. Of note, the current literature has rarely considered the adolescents versus children and/or adults comparison for the evaluation of fatigability. While it seems that children and adolescents have lower level of fatigability and longer TTF than adults, the lack of consistency in the experimental procedures prevent an appropriate interpretation. For instance, while some studies used experimental designs where the exercise duration (or the number of contractions in the case of intermittent exercises) was fixed, e.g., a 30-s sustained maximal voluntary contraction (MVC) (Halin et al., 2003; Streckis et al., 2007; Hatzikotoulas et al., 2009; Gorianovas et al., 2013; Lazaridis et al., 2018), many other studies used protocols with a pre-set amount of fatigability, e.g., the exercise stopped when the subject reached a decrease of 40% of the baseline force level (Armatas et al., 2010; Hatzikotoulas et al., 2014; Piponnier et al., 2018; Piponnier et al., 2019a; Bontemps et al., 2019; Piponnier et al., 2019b; Piponnier et al., 2020), thus preventing any comparison of fatigability between youth and adults. To allow a reliable comparison for the level of fatigability between youth and adults, studies should report an “isotime” measurement that includes only the portion of the fatiguing exercise that is available for all the subjects being analyzed, which is limited by the subject with the shortest TTF (Nicolò et al., 2019). This specific method of analysis allows to compare youth versus adults at a similar exercise duration, without any consideration of the total TTF that could largely differ between these two populations (Armatas et al., 2010; Barker et al., 2010; Hatzikotoulas et al., 2014; Ratel et al., 2015; Piponnier et al., 2019a; Bontemps et al., 2019; Piponnier et al., 2019b).

While various fatiguing protocols have been used to assess either TTF and/or fatigability in youth and adults, one should note that the exercise modality may influence the reported differences in these two concepts when these two populations are compared. First, the type of exercise, i.e., dynamic (e.g., cycling, running, jumping, isokinetic contractions) versus isometric exercises, should be considered when looking at potential between-group differences in fatigability and/or TTF. This issue has not been directly addressed so far and a critical review of the literature could help to better understand how the type of exercise could influence the potential differences in fatigability and TTF between youth and adults. This is an important question since large differences in physiological demands exist between these two types of exercise. For instance, performing either a dynamic or isometric exercise may modulate the influence of blood flow occlusion on exercise performance. Intramuscular pressure is dramatically increased during isometric compared to dynamic exercises (Lind, 2011). This could lead to large variations in the stimulation of type III/IV metabo-nociceptive afferents, which project their inputs to various sites within the central nervous system then modulating exercise performance (Amann et al., 2011; Hureau et al., 2018). The consideration of two populations with fundamental differences in physiological functioning (Ratel and Blazevich, 2017; Patikas et al., 2018) may exacerbate the effect that blood flow occlusion could have on the level of fatigability and TTF depending on the type of exercise that is used. For instance, large differences in muscle mass may have a direct impact on intramuscular pressure and blood flow occlusion during exercise. Second, one may question the influence that exercise duration could have in the differences in TTF and fatigability between youth and adults, i.e., do differences in TTF and/or fatigability between children and adults are greater or lower for short or long-duration exercises? Indeed, exercise duration could influence the mechanisms responsible for impairments in exercise performance (Brownstein et al., 2020). Longer exercise duration usually leads to a larger magnitude of fatigability (e.g., greater loss of maximal force), as reported for instance in running exercises (Temesi et al., 2021). Because the contribution of each energy systems (i.e., aerobic versus anaerobic metabolisms) in exercise performance may differ between youth and adults, it is tempting to suggest that differences in fatigability and TTF between youth and adults may be influenced by exercise duration. Such a question has never been studied so far, and a quantitative analysis of the current literature could help to shed light on this specific point.

The primary purpose of this study is to systematically review the literature regarding the differences in fatigability and TTF between youth (including children and adolescents) and young adults (18–35 years old) and to assess the influence of exercise modalities on these outcomes. The secondary aim of this review is to identify the physiological mechanisms underlying the reported differences between youth and adults, based on neuromuscular evaluation (i.e., central and peripheral components of fatigue).

## 2 Materials and methods

### 2.1 Literature search strategy

We looked for cross-sectional and longitudinal studies that compared TTF and fatigability between youth (i.e., including both children and adolescents) and young adults (18–35 years old). A distinction is made throughout the manuscript between children and adolescents, with the former and the latter referring to prepubescent and pubescent young individuals, respectively. We made this distinction based on objective criteria displayed in the articles, e.g., Tanner classification and/or peak height velocity. When no objective criteria were given, we arbitrary classified the data in the children category (knowing that it could include both prepubescent and pubescent young individuals). We used the following electronic databases: Medline (*via* PubMed), SPORTDiscus and Cochrane Library. Each database was searched from inception until 5 June 2020. The search was conducted by combining terms related to the intervention (e.g., whole-body or isometric exercises), population (e.g., child, adolescent, prepubertal, pubertal) and outcomes (e.g., fatigability, isometric and/or dynamic force, power, endurance, number of contractions, time to exhaustion). There was a language restriction (English or French) and only accepted or published studies were considered. Details of the protocol and search strategy (Supplementary Table S1, Supplemental Digital Content 1, which displayed detailed information about our search strategy) for this systematic review were registered on PROSPERO (CRD42020184549).

### 2.2 Selection of studies

**FIGURE 1:**
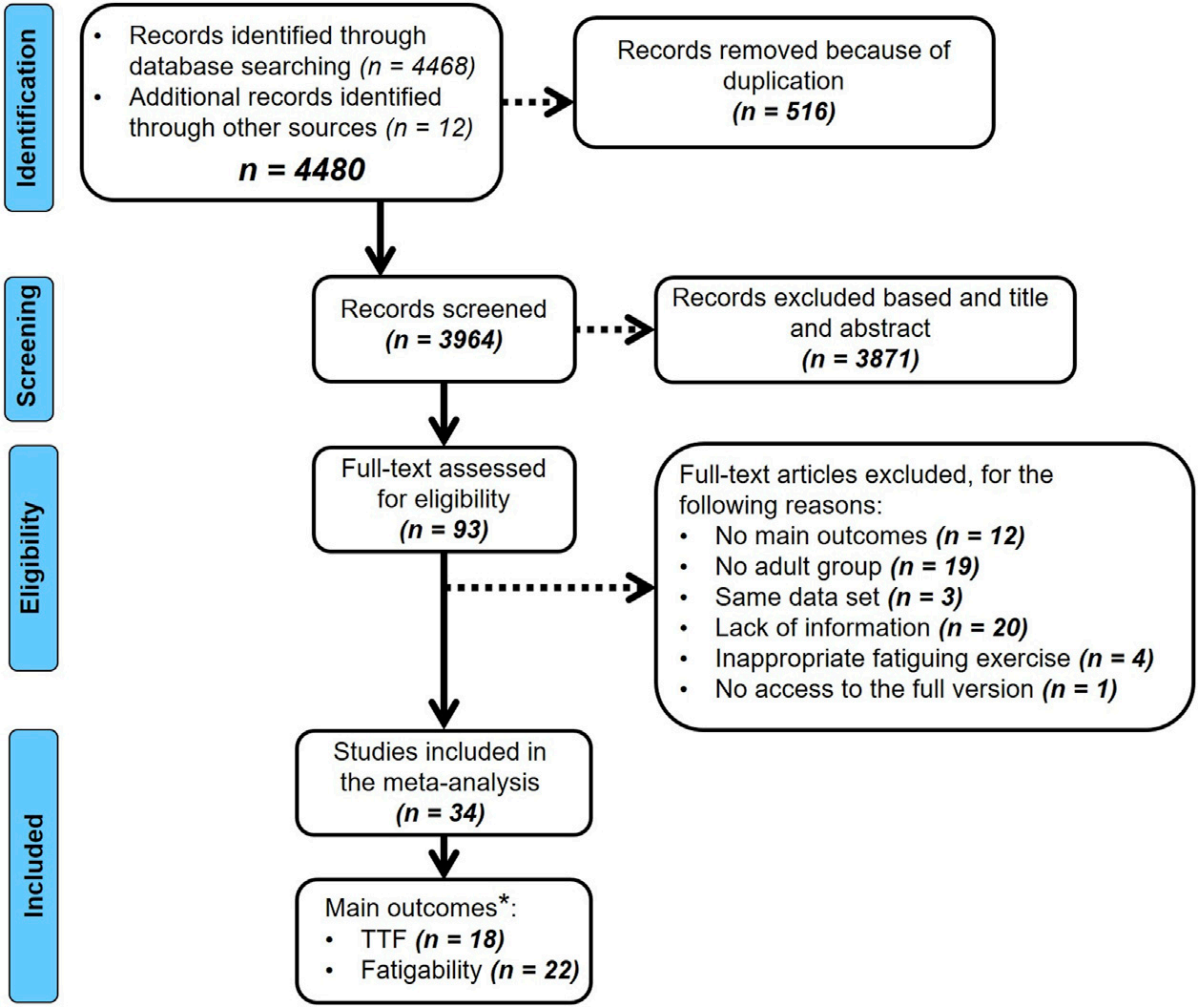
PRISMA flowchart of included studies. *Some articles investigated TTF and fatigability in the same experimental design and were included in both meta-analyses.

The initial search was performed by two authors (RS and MG). The first step consisted in screening titles and abstracts. The articles that were judged to be outside the scope of this meta-analysis were removed. Following this first screening, and in the case the abstracts did not provide enough information, two authors (RS and MG) independently selected and reviewed all included articles. At this point, all duplicate studies were removed. The articles that met the inclusion criteria were read and eligible studies were included in the meta-analyses (Figure 1). Disagreements were solved by a third author (PD).

### 2.3 Eligibility criteria—inclusion and exclusion

Studies were considered for review if they met the following PICOS (i.e., Population, Intervention; Comparison group; Outcomes; Study type) criteria: 1) Comparison between youth (<18 years) and adults (18–35 years); 2) existence of a fatiguing exercise protocol (i.e., dynamic or isometric); 3) assessment of fatigability (i.e., evaluated by changes in muscle force and/or power and/or velocity after the fatiguing exercise) and/or TTF (i.e., evaluated by a TTF in the case of continuous exercises or a number of repetitions in the case of intermittent exercises); 4) cross-sectional and longitudinal studies (in the case of longitudinal studies that assessed the effect of a training intervention, only baseline data were included). The exclusion criteria were 1) a lack of a comparison group (i.e., adult group); 2) an absence of investigation of the main outcomes of interest for the meta-analysis (i.e., isometric or dynamic muscle force, power output, maximal velocity, TTF, number of contractions); 3) an adult group older than 35 years; 4) any publications written in another language than English or French.

### 2.4 Quality assessment

The methodological quality assessment of all studies included in the meta-analysis was performed with a modified Newcastle-Ottawa Quality Assessment Scale for cross-sectional studies. This scale is based on three broad criteria that are specific to the study design, i.e., 1) selection of study groups; 2) comparability and 3) outcome assessments. Similarly to a recent meta-analysis that investigated the differences in fatigability between healthy young and old subjects (Kruger et al., 2018), we modified the original quality scale to meet the needs of our study design (Supplementary Material S2, Supplemental Digital Content 2 which displayed the items for the quality assessment of the included studies). First, the selection domain presents three sub-categories, i.e., the representativeness of the sample (is the sample representative of the average in the target population or was it a sample from selected group of users?), the sample size (is there a justification for sample size calculation?) and the ascertainment of participants’ health status (is the participants’ health status checked with medical report or specific questionnaire or is the participants’ health status only basically reported?). As performed by Kruger et al. (2018), the ascertainment of exposure section from the original scale has been adapted to our study design to have information on participants’ health status. Second, the comparability domain allowed us to control for physical activity and fitness levels and to control for any additional factors that could have impacted the main outcomes (e.g., caffeine and alcohol consumption, strenuous physical activity before physical testing). Third, the outcome domain presents two sub-categories, i.e., the assessment of the main outcomes (are the main outcomes obtained after reliable fatiguing protocol and using validated measuring tools?) and the statistical test (is the statistical test used to analyze the data clearly described and appropriate?). The quality of the paper was rated by stars, ranking from zero to four for the selection domain, zero to two for the comparability domain and zero to three for the outcome domain, for a maximum of nine stars (one being least quality and nine maximum quality). The global quality scores were calculated based on the scoring algorithm proposed by McPheeters et al. (2012).

### 2.5 Data extraction

For all studies included in the meta-analysis, study characteristics (i.e., authors, year, sample size, study design), participant’ demographics (i.e., age, sex), fatiguing exercise details (i.e., isometric or dynamic fatiguing task, muscle(s) involved in the exercise) and main outcomes (i.e., muscle force and/or power and/or velocity, TTF, number of contractions) were retrieved on a standardized Excel sheet. Corresponding authors were contacted when data were missing. Different meta-analyses were performed for the main outcomes (see the sections below).

#### 2.5.1 Time to task failure

The first meta-analyses compared TTF between children versus adults and adolescents versus adults. In the case of sustained prolonged exercises, the total time (in seconds) was extracted for further analysis. In the case of intermittent exercises, and if the article only reported the total number of contractions until task failure, the data were extracted and transformed in time units (i.e., seconds). The resting time allowed between each intermittent contraction (e.g., 5 s ON/5 s OFF) was included in the calculation to obtain the total exercise duration. If these information (i.e., number of contractions, duration of the contraction, resting time allowed between contractions) were missing, the article was not included in these meta-analyses. Further, studies were excluded for TTF-related meta-analysis (but not fatigability analysis, see below) if the exercise was performed with a fixed duration (e.g., a sustained submaximal isometric contraction for 10 min) or a fixed number of contractions (e.g., 50 intermittent contractions with 5 s ON/5 s OFF). When more than one fatiguing exercise was performed in a similar study (e.g., sustained contraction at 20 and 60% MVC), the data obtained during the longest exercise was kept for quantitative analysis.

#### 2.5.2 Performance fatigability

Additional meta-analyses compared the indices of fatigability (i.e., muscle force, power and/or velocity) between children versus adults and adolescents versus adults. The analyses were only performed for data extracted from studies that used an experimental design where the exercise duration or the number of contractions were fixed (e.g., a sustained maximal isometric contraction maintained for 2 min or a similar number of intermittent contractions performed by participants over a similar period of time). When a study did not meet these criteria (e.g., a study where exercise termination was set to a 30% decrease in maximal force, independently of the time to reach this target), the authors were contacted to know if they performed an “isotime” comparison, i.e., the analysis that includes only the portion of the exercise that is available for all the participants in the groups being analyzed, which is limited by the participant with the shortest TTF (Nicolò et al., 2019), or if they were willing to perform such analyses. In such case, these data were included in the meta-analyses. Otherwise, the article was excluded from the meta-analyses. Because huge differences exist in baseline maximal muscle force and power between children and adults, these meta-analyses solely included the studies that reported relative data (i.e., when the changes at the end of the fatiguing exercise were expressed as a percentage of the baseline value). When only absolute data (i.e., Newton for muscle force and Watts for muscle power) were reported, we contacted the authors and asked them to provide the relative data.

#### 2.5.3 Exercise modalities

Separate subgroup meta-analyses were performed to investigate the potential influence of exercise modality on the reported differences in TTF and fatigability between children and adults. For that purpose, we dissociated the type of exercises that were performed, i.e., isometric versus dynamic (e.g., running, cycling, jumping, isokinetic contraction). We also investigated the potential influence of exercise duration on child-adult differences in TTF and fatigability. For this purpose, the median was calculated and the studies were classified either as long (i.e., duration > median) or short (i.e., duration < median) duration.

#### 2.5.4 Peripheral and central components of fatigue

When available, data on central and peripheral factors of fatigue were also extracted as secondary outcomes to shed light on the potential differences in TTF and fatigability between children and adults. The following parameters were considered to investigate central factors of fatigue: 1) Voluntary activation level (VA), consisting in an electrical/magnetic stimulation superimposed to a maximal voluntary contraction (both the interpolated twitch technique and the central activation ratio were considered), 2) normalized electromyographic (EMG) signals recorded during a maximal force; 3) transcranial magnetic stimulation (TMS)-related parameters, i.e., motor evoked potentials (MEP, amplitude and/or area) and silent period duration to investigate corticospinal excitability and inhibition, respectively; 4) H- and T-reflexes to investigate spinal excitability. The following parameters were considered to investigate peripheral factors of fatigue: 1) peak twitch (Pt) and doublet (Db), i.e., the mechanical response to a single or double electrical and/or magnetic stimulation, with its associated characteristics (e.g., half relaxation time, time to Pt) also being considered; 2) M-wave amplitude and/or area, i.e., the EMG response to a single electrical and/or magnetic stimulation; 3) low-to-high frequency fatigue ratio (LHF_R_), i.e., the ratio of peak forces evoked by low and high-frequency doublets or stimulation trains (e.g., the ratio between Db evoked at 10 and 100 Hz).

### 2.6 Data analysis

Descriptive statistics (mean, median, range) were used to describe studies characteristics and methodological quality of all the included studies.

Hedges’ g were calculated (Hedges and Olkin, 1985) as the measure of standardized mean difference (SMD), i.e., the difference between the outcome mean values of the children or adolescents and the adult group divided by the pooled standard deviation (Hedges and Olkin, 1985; Morris and DeShon, 2002). A negative SMD indicates less fatigability or TTF in children, whereas a positive SMD represents greater fatigability or TTF in adults. To assess the difference in outcomes of interest between children and adults, effect sizes were estimated by weighting SMDs by the inverse of their variance based on random effects models (Shadish and Haddock, 2009).

Heterogeneity was tested with Cochran’s chi-square test (Q) to assess the consistency of associations. To quantify the extent of heterogeneity, we estimated the between-study variance (I^2^). I^2^ statistic describes the proportion of variability in SMDs due to the heterogeneity between studies ranging from 0% to 100% (with small heterogeneity: < 25%; moderate: 25–50%; high: ≥ 50%). Because heterogeneity was high (I^2^ > 50%), random effect models were used to incorporate heterogeneity in meta-analyses (Higgins et al., 2003).

In addition, effect sizes were computed for subgroups of included studies based on dichotomized identified exercise modalities that may impact our main outcomes, i.e., type (isometric versus dynamic) and duration of exercise (< median versus ≥ median duration across studies). Publication bias was searched by funnel plot representation and Egger’s (Egger et al., 1997) and Begg’s (Begg and Mazumdar, 1994) tests with *p* < 0.10 taken as an indication of publication bias. All statistical analyses were carried out by using Stata software version 11 (StataCorp, College Station, TX, United States).

## 3 Results

The process of study identification, screening, and evaluation of the eligibility of included studies is displayed by the PRISMA flow chart (Figure 1). The initial searches provided a total of 4,468 articles. Following the removal of duplicates, the titles and abstracts of the remaining 3,964 records were screened, with 3,871 being excluded at this stage for not meeting the inclusion criteria. Then, full texts of 93 records were assessed for eligibility with a further 59 of these being removed for various reasons (i.e., lack of main outcomes, lack of adult group, same data set, inappropriate fatiguing exercise, no access to the full version of the article, lack of information that includes for instance studies reported only absolute rather than relative data for the fatigability domain). This leaves 34 records that were included in the meta-analyses. A detailed description of the characteristics of the meta-analyzed studies that investigated differences in TTF and fatigability between children, adolescents and adults is given in Tables 1, 2, respectively.

**TABLE 1:** Study characteristics—Fatigability.

Study	Participant’s age (*n*)			Fatiguing exercise (*criteria for exercise ending*)	Main outcome (investigated muscle)	% decrease		
	Ch	Ado	Adu			Ch	Ado	Adu	Statistical significance
**ISOMETRIC EXERCISE**									
Bontemps et al. (2019)	10 (18)	—	22 (19)	5 s MVC/5 s rest (*↓ of 40% MVC*)	I_MVC_ (KE)	**38**	**—**	**49**	S
Halin et al. (2003)	11 (15)	—	22 (12)	30-s sustained MVC	I_MVC_ (EF)	**29**	**—**	**35**	S
Hatzikotoulas et al. (2009)	10 (15)	—	24 (15)	10-min sustained 20% MVC	I_MVC_ (PF)	**24**	**—**	**26**	NS
Piponnier et al. (2020)	10 (19)	—	22 (23)	5 s MVC/5 s rest optimal length (*↓ of 40% MVC*)	IMVC (PF)	**36**	**—**	**22**	S
				5 s MVC/5 s rest long length (*↓ of 40% MVC*)		**34**	**—**	**25**	S
				5 s MVC/5 s rest short length (*↓ of 40% MVC*)		**35**	**—**	**26**	S
Piponnier et al. (2019b)	10 (22)	—	21 (22)	5 s MVC/5 s rest optimal length (*↓ of 40% MVC*)	I_MVC_ (KE)	**25**	**—**	**34**	S
				5 s MVC/5 s rest long length (*↓ of 40% MVC*)		**27**	**—**	**23**	NS
				5 s MVC/5 s rest short length (*↓ of 40% MVC*)		**24**	**—**	**22**	NS
Piponnier et al. (2019a)	10 (9)	14 (8)	24 (11)	5 s MVC/5 s rest (*↓ of 40% MVC*)	I_MVC_ (KE)	**20**	**25**	**32**	S
Ratel et al. (2015)	10 (11)	—	24 (12)	5 s MVC/5 s rest (*↓ of 40% MVC*)	I_MVC_ (KE)	**22**	**—**	**30**	S
Willcocks et al. (2014)	—	13 (6)	29 (6)	30 × 6 s MVC	I_MVC_ (KE)	**—**	**23**	**31**	NS
**DYNAMIC EXERCISE**									
Äyrämö et al. (2017)	12 (8)	15 (8)	21 (8)	50-s all out run	I_MVC_ (PF)	**3**	**+7**	**16**	NS (Ch vs. Adu) S (Ado vs. Adu)
Birat et al. (2018)	10 (12)	—	21 (12)	30-s Wingate test	MP (LL)	**35**	**—**	**52**	S
Buchheit et al. (2010)	10 (10)	15 (6)	20 (7)	10 × 10-s all-out cycling sprint	MP (LL)	**6**	**6**	**5**	NS
De Ste Croix et al. (2009)	12 (16)	–	30 (24)	50 CONC MVC	D_MVC_ (KE)	**39**	**—**	**60**	S
Dipla et al. (2009)	11 (10)	15 (10)	24 (10)	4 × 18 KE-KF CONC MVC	D_MVC_ (KE)	**9**	**16**	**29**	S (Ch & Ado vs. Adu)
					D_MVC_ (KF)	**NC**	**15**	**23**	S (Ch vs. Ado & Adu)
Ftikas et al. (2010)	10 (11)	—	24 (11)	10 × 10 max vertical jumps	I_MVC_ (KE)	**13**	**—**	**18**	S
Gorianovas et al. (2013)	12 (11)	—	21 (11)	100 max drop jumps	I_MVC_ (KE)	**22**	**—**	**36**	NR
Hebestreit et al. (1993)	10 (8)	—	22 (8)	30-s Wingate test	MP (LL)	**44**	**—**	**52**	S
Kanehisa et al. (1995)	—	14 (26)	18–25 (27)	50 CON MVC	D_MVC_ (KE)	**—**	**36**	**48**	S
Lazaridis et al. (2018)	10 (13)	—	25 (13)	10 × 10 max CMJ	I_MVC_ (KE)	**12**	**—**	**18**	S
Liamopoulou et al. (2015)	10 (12)	—	25 (12)	10 × 10 max plyometric jumps	I_MVC_ (KE)	**14**	**—**	**22**	S
Marginson et al. (2005)	10 (10)	—	22 (10)	8 × 10 max plyometric jumps	I_MVC_ (KE)	**14**	**—**	**26**	S
Pullinen et al. (2011)	—	14 (6)	27 (6)	5 × 10 contractions 40% RM	I_MVC_ (KE)	**—**	**15**	**25**	NS
Weinstein et al. (2018)	10 (11)	—	20 (10)	30-s Wingate test	MP (LL)	**33**	**—**	**47**	S

Data presented in bold black were given as relative results (PRE-POST, changes in % of PRE) in the published article. Data presented in bold blue were calculated by the authors from the absolute results given in the article. In the cases where data were not fully presented in the manuscript, data were extracted from original figures using ImageJ software (ImageJ V.1.45 s, National Institute of Health, MD, United States). Ado, adolescents; Adu, adult; BP, bench press; Ch, children; CON, concentric; D_MVC_, dynamic maximal voluntary contraction; ECC, eccentric; EF, elbow flexors; I_MVC_, isometric maximal voluntary contraction; KE, knee extensors; KF, knee flexors; LL, lower limbs; MP, muscle power; MVC, maximal voluntary contraction; n, number of participants; NC, no change; NR, not reported; NS, nonsignificant (i.e., *p* > 0.05); PF, plantar flexors; RM, maximum repetition; S, statistically significant (i.e., *p* < 0.05).

**TABLE 2:** Study characteristics—Time to task failure.

Study	Participant’s age (*n*)			Fatiguing exercise (*criteria for exercise ending*)		TTF (in s)		
	Ch	Ado	Adu		Muscle group	Ch	Ado	Adu	Statistical significance
**ISOMETRIC EXERCISE**									
Armatas et al. (2010)	10 (13)	—	26 (13)	5 s MVC/5 s rest (*↓ of 50% MVC*)	KE	**563**	—	**348**	S
Bontemps et al. (2019)	10 (18)	—	22 (19)	5 s MVC/5 s rest (*↓ of 40% MVC*)	KE	**404**	—	**159**	S
Hatzikotoulas et al. (2014)	11 (10)	—	26 (11)	Sustained MVC (*↓ of 50% MVC*)	PF	**127**	—	**94**	S
Patikas et al. (2013)	10 (14)	—	24 (14)	Sustained 20% MVC (5 *s < 95% target force*)	PF	**771**	—	**786**	NS
				Sustained 60% MVC (5 *s < 95% target force*)		**195**	—	**201**	NS
Piponnier et al. (2020)	10 (19)	—	22 (23)	5 s MVC/5 s rest optimal length (*↓ of 40% MVC*)	PF	**156**	—	**135**	NS
				5 s MVC/5 s rest long length (*↓ of 40% MVC*)		**120**	—	**130**	NS
				5 s MVC/5 s rest short length (*↓ of 40% MVC*)		**170**	—	**160**	NS
Piponnier et al. (2019b)	10 (22)	—	21 (22)	5 s MVC/5 s rest optimal length (*↓ of 40% MVC*)	KE	**397**	—	**148**	S
				5 s MVC/5 s rest long length (*↓ of 40% MVC*)		**295**	—	**158**	S
				5 s MVC/5 s rest short length (*↓ of 40% MVC*)		**337**	—	**409**	NS
Piponnier et al. (2019a)	10 (9)	14 (8)	24 (11)	5 s MVC/5 s rest (*↓ of 40% MVC*)	KE	**529**	**426**	**266**	S (Ch & Ado vs. Adu)
Ratel et al. (2015)	10 (11)	—	24 (12)	5 s MVC/5 s rest (*↓ of 40% MVC*)	KE	**495**	—	**340**	S
Tanina et al. (2017)	9 (14)	—	25 (14)	Sorensen back test (*> 2 cm reduction in height for* 2 *s*)	TE	**95**	—	**98**	NS
Woods et al. (2019)	10 (18)	—	24 (21)	Intermittent 5 s submaximal contractions (*volitional exhaustion*)	KE	**688**	—	**632**	NS
Woods et al. (2020)	10 (17)	—	24 (17)	Intermittent 5 s submaximal contractions (*volitional exhaustion*)	KE	**608**	—	**560**	NS
**DYNAMIC EXERCISE**									
Bar Yoseph et al. (2019)	11 (18)	17 (18)	29 (8)	Incremental cycling exercise (*cadence < 60 rpm*)	LL	**670**	**692**	**690**	NS
				Incremental running exercise (*volitional exhaustion*)		**700**	**951**	**1,013**	S (Ado & Adu vs. Ch)
Berthoin et al. (2003)	11 (9)	—	22 (8)	Cycling at 120% PMA (*volitional exhaustion*)	LL	**53**	—	**122**	S
Leclair et al. (2011)	10 (15)	—	24 (15)	Constant load cycling exercise P50 (*cadence < 70 rpm*)	LL	**702**	—	**754**	NS
				Constant load cycling exercise P75 (*cadence < 70 rpm*)		**307**	—	**371**	
				Constant load cycling exercise P100 (*cadence < 70 rpm*)		**144**	—	**221**	S
				Constant load cycling exercise P110 (*cadence < 70 rpm*)		**96**	—	**147**	S
Murphy et al. (2014)	10 (10)	—	26 (10)	3 × max CON MVC Low RM (*volitional exhaustion*)	KE	**274**	—	**253**	S
				3 × max CON MVC High RM (*volitional exhaustion*)		**213**	—	**193**	
Pullinen et al. (2011)	—	14 (8)	31 (8)	3 × max contractions at 40% RM (*volitional exhaustion*)	KE	—	**48**	**48**	NS
Pullinen et al. (2002)	—	14 (6)	27 (6)	1 × max contractions at 40% RM (*volitional exhaustion*)	KE	—	**46**	**42**	NS
Tibana et al. (2012)	—	15 (15)	22 (15)	3 × max chest press w/30 s rest (*volitional exhaustion*)	UL	—	**96**	**88**	S
				3 × max chest press w/60 s rest (*volitional exhaustion*)		—	**163**	**156**	S
				3 × max chest press w/120 s rest (*volitional exhaustion*)		—	**292**	**282**	S

Data presented in bold black were directly given in time units (seconds) in the article. Data presented in bold blue were calculated by the authors from the number of contractions performed during the fatiguing exercise. Ado, adolescents; Adu, adult; Ch, children; CON, concentric; KE, knee extensors; KF, knee flexors; LL, lower limbs; MVC, maximal voluntary contraction; n, number of participants; NS, nonsignificant; P50 and P75, intensities corresponded to 50 and 75% of the difference between maximal aerobic power and the power associated with the ventilatory threshold; P100 and P110, intensities corresponded to 100 and 110% of maximal aerobic power; PF, plantar flexors; RM, maximum repetition; S, statistically significant (i.e., *p* < 0.05); TE, trunk extensors; TTF, time to task failure; UL, upper limbs.

### 3.1 Quality assessment

Studies that met the inclusion criteria ranged between two and seven stars (one being least quality and nine maximum quality), with a mean score of 4.3 ± 1.2 and a median of 4 (Supplementary Table S3, Supplemental Digital Content 3 which presents the detailed results for quality assessment of the included studies). Considering the classification proposed by McPheeters et al. (2012) and regarding the risk of bias, 0% (0/29), 28% (8/29) and 72% (21/29) of the included studies had a good, fair and poor grade, respectively. For the comparability domain, 21% (6/29), 55% (16/29) and 24% (7/29) of the studies had a good, fair and poor grade, respectively. Finally, for the outcome domain, 17% (5/29), 76% (22/29) and 7% (2/29) of the studies had a good, fair and poor grade, respectively.

### 3.2 Publication bias

Regarding TTF, no evidence of publication bias was identified by Begg’s and Egger’s tests (*p* > 0.10) or funnel plot representation (Supplementary Figure S1, Supplemental Digital Content 4). However, Begg’s and Egger’s tests indicated evidence for small study-effects in fatigability with *p* = 0.030 and 0.025, respectively. Funnel plot representation (Supplementary Figure S2, Supplemental Digital Content 5) showed a little asymmetry with a few studies with relatively small sample size reporting the largest effects on fatigability in favor of children as compared to adults. A risk of publication bias for fatigability suggests that our analyses may be biased in the sense of an overestimation of the fatigability differences between children and adults.

### 3.3 Time to task failure

**FIGURE 2:**
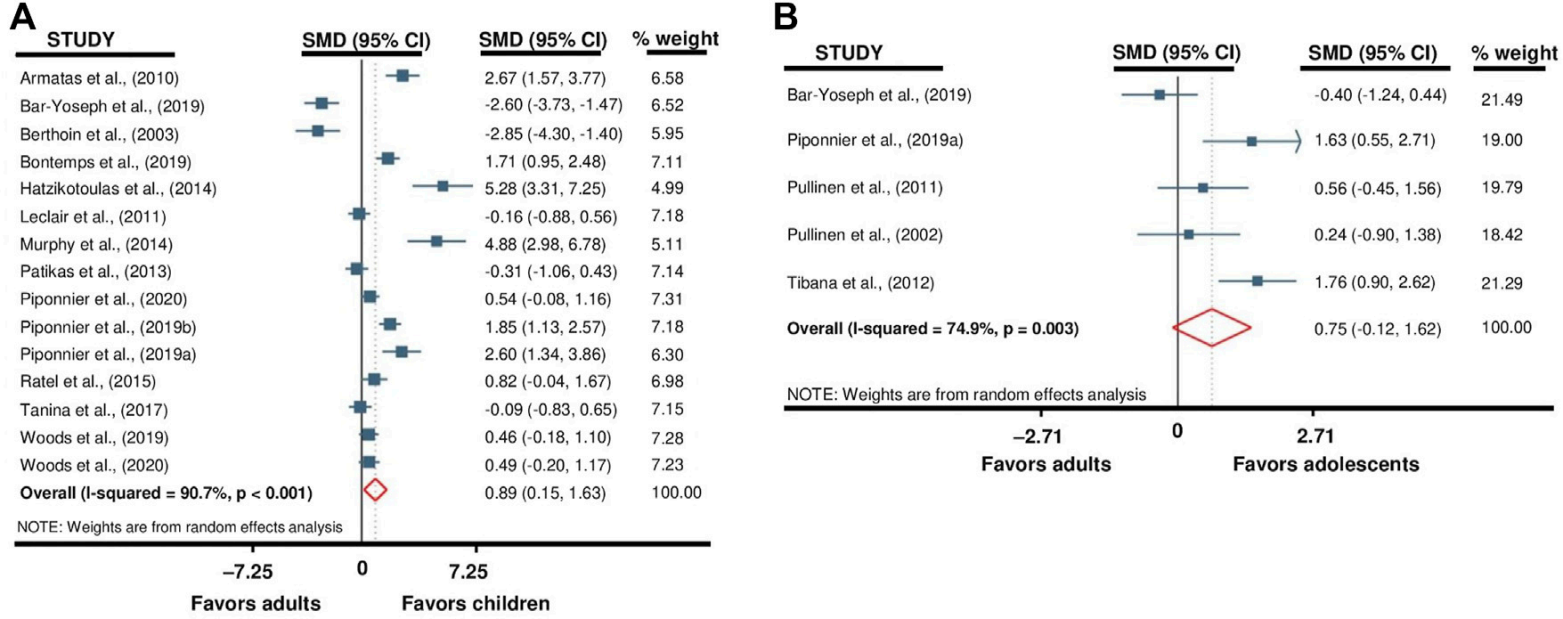
Forest plot from the meta-analysis reporting TTF differences between children versus adults **(A)** and adolescents versus adults **(B)**.

Two separate meta-analyses compared TTF between children versus adults and adolescents versus adults. The exercise duration of 12 out of 18 (67%) of the studies included in these meta-analyses (Pullinen et al., 2002; Berthoin et al., 2003; Armatas et al., 2010; Pullinen et al., 2011; Tibana et al., 2012; Murphy et al., 2014; Ratel et al., 2015; Piponnier et al., 2019a; Piponnier et al., 2019b; Woods et al., 2019; Piponnier et al., 2020; Woods et al., 2020) was derived from the total number of contractions performed until task failure. The TTF in seconds was directly provided in the other six studies (Berthoin et al., 2003; Leclair et al., 2011; Patikas et al., 2013; Hatzikotoulas et al., 2014; Tanina et al., 2017; Bar-Yoseph et al., 2019). The meta-analyses indicated that TTF was longer in children when compared to adults (SMD 0.89; 95% CI 0.15 to 1.63; *p* = 0.018; *15 studies*, *n = 435*; Figure 2A), with no differences for the adolescents *versus* adults comparison (SMD 0.75; 95% CI –0.12 to 1.62; *p* = 0.090; *5 studies, n = 103*; Figure 2B). Heterogeneity of the results was high for children (Q = 150.5; df = 14; *p* < 0.001; I^2^ = 90.7%) and adolescents (Q = 15.9; df = 4; *p* = 0.003; I^2^ = 74.9%) versus adults comparison.

**FIGURE 3:**
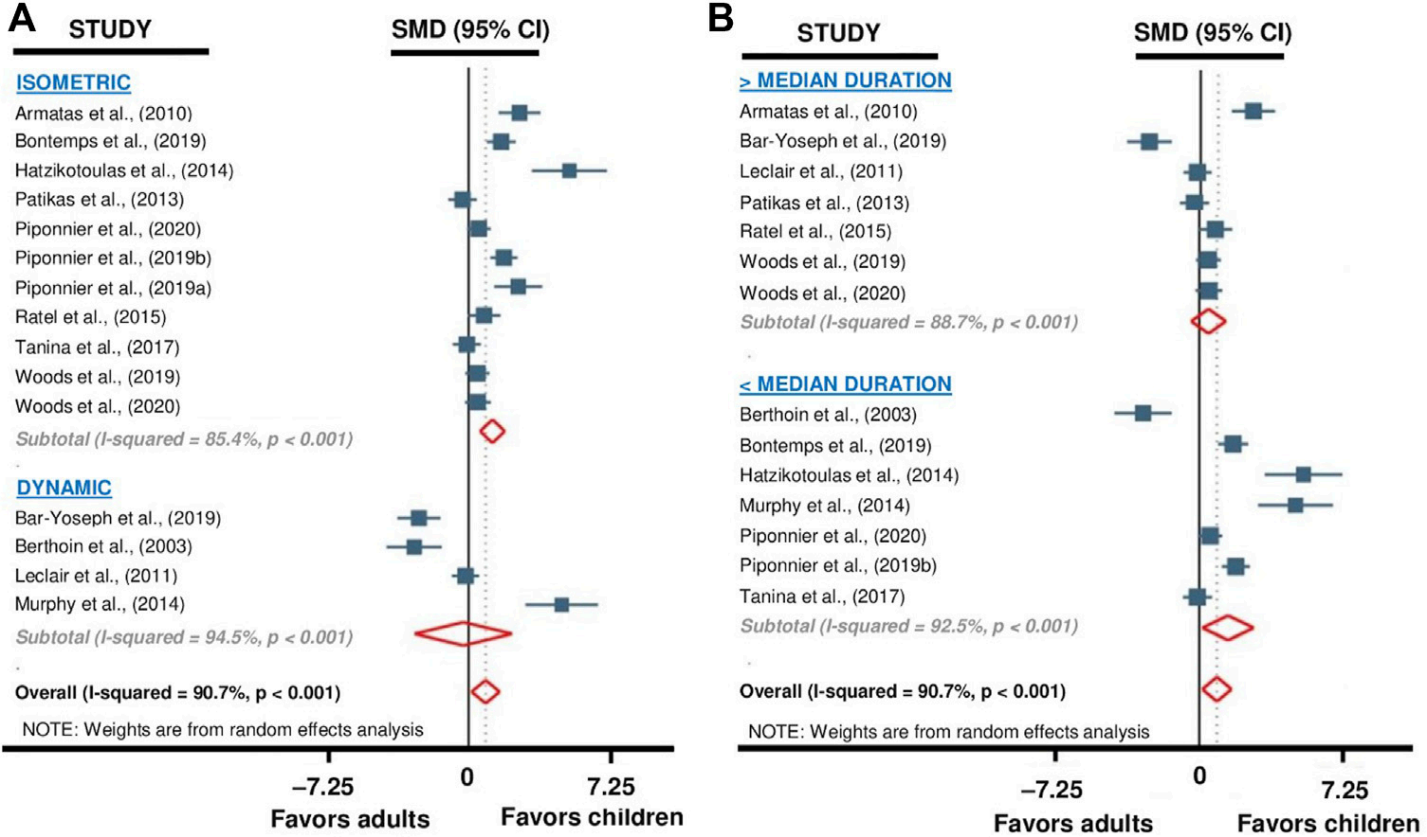
Forest plot from the subgroup meta-analysis reporting the influence of 1) exercise modality (i.e., isometric or dynamic; **(A)** and 2) exercise duration **(B)** on TTF differences between children and adults. Raw SMDs (with 95% confidence intervals) and % weights of the meta-analyzed studies are given in Figure 2.

A first subgroup meta-analysis was performed to evaluate the influence of the type of exercise (i.e., isometric versus dynamic) on the reported differences in TTF between children and adults. This analysis revealed that children had longer TTF than adults for isometric exercises (SMD 1.25; 95% CI 0.60 to 1.90; *p* < 0.001; *11 studies*, *n = 342*; Figure 3A), but no difference was found for dynamic exercises (SMD −0.27; 95% CI −2.82 to 2.28; *p* = 0.83; *4 studies*, *n = 93*; Figure 3A). The heterogeneity of the results obtained for subgroup isometric (Q = 68.6; df = 10; *p* < 0.001; I^2^ = 85.4%) and dynamic (Q = 54.5; df = 3; *p* < 0.001; I^2^ = 94.5%) exercises analysis was high. Because of an unbalanced repartition of studies (i.e., one with isometric and four with dynamic modality of exercise), this subgroup analysis was not performed for the adolescents versus adults comparison.

A second subgroup meta-analysis was performed to evaluate the influence of exercise duration on the reported differences in TTF between children and adults. For this analysis, the study with the duration equal to the median (Piponnier et al., 2019a) was excluded, thus 14 studies were included. The mean duration (pooled data of children and adults participants) of the studies in the short-duration and the long-duration categories were 180 ± 89 s (range: 88–282 s) and 640 ± 164 s (range: 418–857 s), respectively. This analysis indicated that differences in TTF were significant between children and adults for short-duration exercises (SMD 1.46; 95% CI 0.16 to 2.76; *p* = 0.028; *7 studies, n = 209*; Figure 3B) while it was not for long-duration ones (SMD 0.20; 95% CI –0.66 to 1.07; *p* = 0.64; *7 studies, n = 206*; Figure 3B). The heterogeneity of the results obtained for the subgroup analysis relative to short- (Q = 80.4; df = 6; *p* < 0.001; I^2^ = 92.5%) and long-duration (Q = 48.9; df = 6; *p* < 0.001; I^2^ = 87.7%) exercises was high. Because of a too low number of studies (i.e., two versus two studies if the median was excluded), this subgroup analysis was not performed for the adolescents versus adults comparison.

### 3.4 Fatigability

**FIGURE 4:**
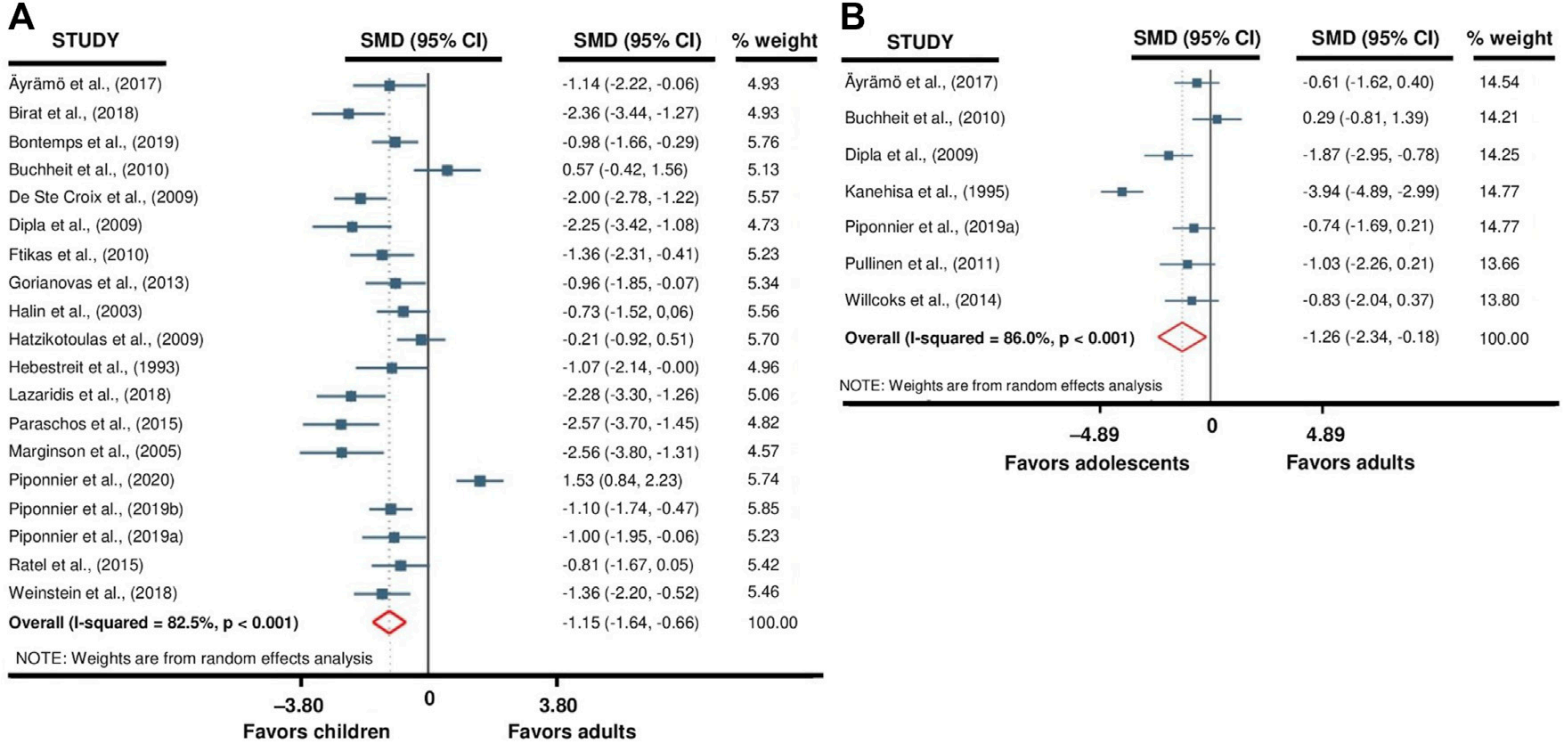
Forest plot from the meta-analysis reporting differences in fatigability between children versus adults **(A)** and adolescents versus adults **(B)**.

The meta-analyses that aimed to evaluate the differences in fatigability between children versus adults and adolescents versus adults included 19 and 7 studies, respectively. The analyses revealed that children (SMD −1.15; 95% CI −1.64 to –0.66; *p* < 0.001; *19 studies*, *n = 489*; Figure 4A) and adolescents (SMD −1.26; 95% CI −2.34 to −0.18; *p* = 0.022; *7 studies*, *n = 149*; Figure 4B) were significantly less fatigable when compared to adults, with the heterogeneity of studies being high either for the children versus adults (Q = 103.1; df = 18; *p* < 0.001; I^2^ = 82.5%) or adolescents versus adults (Q = 42.8; df = 6; *p* < 0.001; I^2^ = 86.0%) comparisons.

**FIGURE 5:**
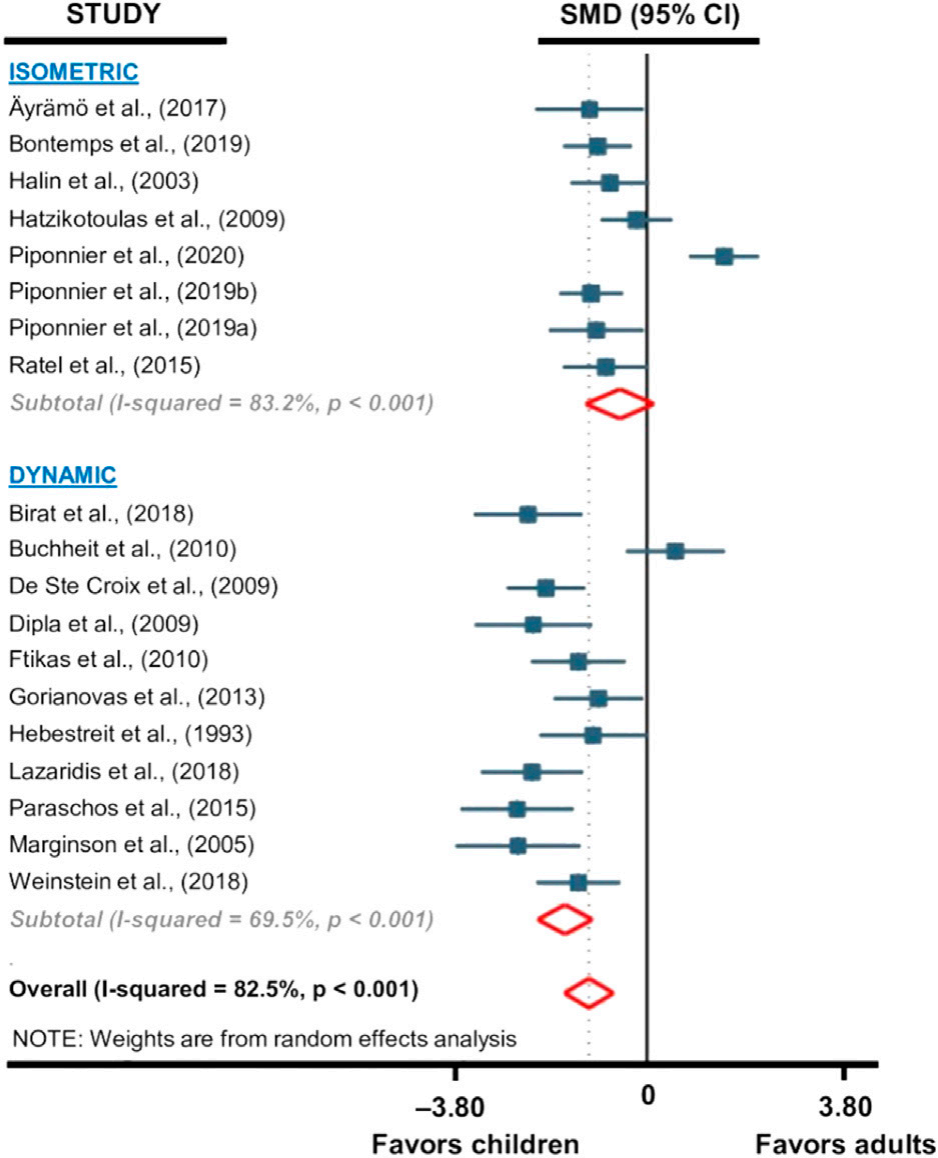
Forest plot from the subgroup meta-analysis reporting the influence of exercise modality (i.e., isometric or dynamic) on fatigability differences between children and adults. Raw SMDs (with 95% confidence intervals) and % weights of the meta-analyzed studies are given in Figure 4.

A subgroup analysis was performed to evaluate the influence of the type of exercise on the reported differences in fatigability between children and adults. This subgroup analysis was not performed on adolescents because of a too low number of studies. The subgroup meta-analysis reported that children were significantly less fatigable than adults during dynamic exercises (SMD −1.58; 95% CI −2.08 to −1.08; *p* < 0.001; *12 studies, n = 275*; Figure 5), but non-significant difference was found for isometric exercises (SMD –0.46; 95% CI −1.19 to 0.27; *p* = 0.22; *7 studies*, *n = 214*; Figure 5). The heterogeneity of the studies involved in isometric (Q = 41.8; df = 7; *p* < 0.001; I^2^ = 83.2%) and dynamic (Q = 32.7; df = 10; *p* < 0.001; I^2^ = 69.5%) exercises subgroups analyses was high. Because of too low number of studies (i.e., three *versus* four studies), this subgroup analysis was not performed for the adolescents versus adults comparisons.

### 3.5 Secondary outcomes

Out of the 34 studies included in the meta-analyses, eight (Hatzikotoulas et al., 2014; Murphy et al., 2014; Ratel et al., 2015; Äyrämö et al., 2017; Piponnier et al., 2019a; Bontemps et al., 2019; Piponnier et al., 2019b; Piponnier et al., 2020) used neurostimulation techniques to shed light on the central and peripheral factors that may explain the differences in TTF and fatigability between children and adults. Two additional studies (Streckis et al., 2005; Streckis et al., 2007) that were not included in the previous meta-analyses (i.e., because relative data have not been provided by the authors) also investigated the central and peripheral components of fatigability in both children and adults after exercise. Detailed information on these studies is given in Table 3. Because of the small number of studies and the large heterogeneity in the methods used to investigate the peripheral and central factors underlying fatigability, no meta-analyses were performed.

**TABLE 3:** Central and peripheral fatigue parameters.

	Fatiguing exercise (*criteria for exercise ending*)	Central factors			Peripheral factors					
		VA/CAR	EMG/M-wave	H-reflex	Pt	HRT	TPT	M-wave	Db	LHF_R_
Äyrämö et al. (2017)	50-s all out run	**—**	↑ Adu = Ch	↑ Adu = Ch	↓ Adu > Ch	↓ Adu = Ch	↓ Adu = Ch	↓ Adu = Ch	**—**	**—**
Bontemps et al. (2019)	5 s MVC/5 s rest (*↓ of 40% MVC*)	↓ Ch > Adu	↓ Ch > Adu	**—**	↓ Adu > Ch	**—**	**—**	NC	↓ Adu > Ch	↓ Adu > Ch
Hatzikotoulas et al. (2014)	Sustained MVC (*↓ of 50% MVC*)	↓ Ch = Adu	**—**	**—**	↓ Adu > Ch	**—**	↓ Adu > Ch	↓ Adu = Ch	**—**	**—**
Murphy et al. (2014)	3 × max CON MVC (*volitional exhaustion*)	**—**	**—**	**—**	↓ Adu > Ch	↑ Adu > Ch	**—**	↓ Adu > Ch	**—**	**—**
Piponnier et al. (2020)	5 s MVC/5 s rest (*↓ of 40% MVC*)	↓ Ch > Adu	↓ Ch = Adu	↓ Ch = Adu	↓ Adu > Ch	**—**	**—**	↑ Adu > Ch	↓ Adu > Ch	↓ Adu > Ch
Piponnier et al. (2019b)	5 s MVC/5 s rest (*↓ of 40% MVC*)	↓ Ch > Adu	↓ Ch > Adu	**—**	↓ Adu > Ch	**—**	**—**	NC	↓ Adu > Ch	**—**
Piponnier et al. (2019a)	5 s MVC/5 s rest (*↓ of 40% MVC*)	↓ Ch > Adu	↓ Ch > Adu	**—**	↓ Adu > Ch	**—**	**—**	NC	**—**	**—**
					↓ Adu > Ado					
Ratel et al. (2015)	5 s MVC/5 s rest (*↓ of 40% MVC*)	↓ Ch > Adu	NC	**—**	↓ Adu > Ch	**—**	**—**	NC	**—**	**—**
Streckis et al. (2007)	2-min sustained MVC	↓ Ch > Adu	**—**	**—**	↓ Adu > Ch	**—**	**—**	**–**	**—**	**—**
Streckis et al. (2005)	100 drops jumps	**—**	**—**	**—**	↓ Adu > Ch	**—**	**—**	**–**	**—**	↓ Adu > Ch

Ado, adolescents; Adu, adult; CAR, central activation ratio; Ch, children; CON, concentric; Db, doublet; EMG, electromyography; HRT, half relaxation time; LHF_R_, low-to-high frequency fatigue ratio; MVC, maximal voluntary contraction; Pt, peak twitch; TPT, time to peak twitch; VA, voluntary activation level.

#### 3.5.1 Central fatigue

Eight out of the ten studies investigated central fatigue, either using VA (Streckis et al., 2007; Hatzikotoulas et al., 2014; Ratel et al., 2015; Piponnier et al., 2019a; Bontemps et al., 2019; Piponnier et al., 2019b; Piponnier et al., 2020) or the EMG/M-wave ratio (Äyrämö et al., 2017; Piponnier et al., 2019a; Bontemps et al., 2019; Piponnier et al., 2019b; Piponnier et al., 2020) or H-reflexes (Äyrämö et al., 2017; Piponnier et al., 2020). Six studies reported that VA was significantly more decreased at the end of the fatiguing exercise in children than in adults (Streckis et al., 2007; Ratel et al., 2015; Piponnier et al., 2019a; Bontemps et al., 2019; Piponnier et al., 2019b; Piponnier et al., 2020), while one study reported no significant differences in VA changes with fatigue between children and adults (Hatzikotoulas et al., 2014). Three studies reported that the decrease in the EMG/M-wave ratio was significantly more pronounced in children than adults (Piponnier et al., 2019a; Bontemps et al., 2019; Piponnier et al., 2019b). Two studies reported that changes in EMG/M-wave ratio were similar after the exercise between the two populations (Äyrämö et al., 2017; Piponnier et al., 2020), while one study reported no changes in EMG-M-wave ratio after fatigue for both children and adults (Ratel et al., 2015). Finally, two studies reported similar changes in H-reflexes after fatigue in children and adults (Äyrämö et al., 2017; Piponnier et al., 2020).

#### 3.5.2 Peripheral fatigue

All studies reported that Pt and Db were significantly more decreased after exercise in adults compared to children (Streckis et al., 2005; Streckis et al., 2007; Hatzikotoulas et al., 2014; Murphy et al., 2014; Ratel et al., 2015; Äyrämö et al., 2017; Piponnier et al., 2019a; Bontemps et al., 2019; Piponnier et al., 2019b; Piponnier et al., 2020). One study found a greater increase in the half relaxation time (measured from single nerve stimulation) in adults compared to children after exercise (Murphy et al., 2014) while another reported similar changes (Äyrämö et al., 2017). Two studies investigated the time to peak twitch, with one reporting a greater decrease in adults after exercise (Hatzikotoulas et al., 2014) and the other one reporting similar changes between the two populations (Äyrämö et al., 2017). Eight studies investigated M-wave changes with fatigue. One study reported that M-wave decreased more in adults compared to children after exercise (Murphy et al., 2014), while another study reported that M-wave increased in adults after fatigue while it was unchanged in children (Piponnier et al., 2020). The other studies either reported a similar decrease in M-wave between children and adults (Hatzikotoulas et al., 2014; Äyrämö et al., 2017) or no changes in this parameter in both populations (Ratel et al., 2015; Piponnier et al., 2019a; Bontemps et al., 2019; Piponnier et al., 2019b; Piponnier et al., 2020). Lower low-frequency fatigue was found in children compared to adults (Streckis et al., 2005; Bontemps et al., 2019; Piponnier et al., 2020).

## 4 Discussion

This meta-analysis reveals that children have longer TTF and are less fatigable when compared to adults. Complementary analysis reveals that exercise modality (i.e., exercise duration and type of exercise) influences the differences reported in TTF and fatigability between children and adults. While this review points out the lack of studies that investigated differences in TTF and fatigability between adolescents *versus* children and adults, the meta-analyses conducted on available data reported a higher fatigability in adults but no differences in TTF between these two populations.

### 4.1 Children have higher endurance and lower fatigability when compared to adults

Longer TTF were reported in children for ten out of the 15 included studies (Armatas et al., 2010; Hatzikotoulas et al., 2014; Murphy et al., 2014; Ratel et al., 2015; Piponnier et al., 2019a; Bontemps et al., 2019; Piponnier et al., 2019b; Woods et al., 2019; Piponnier et al., 2020; Woods et al., 2020). Seventeen out of the 19 studies that compared fatigability between children and adults observed a lower level of fatigability in children (Hebestreit et al., 1993; Halin et al., 2003; Marginson et al., 2005; De Ste Croix et al., 2009; Dipla et al., 2009; Hatzikotoulas et al., 2009; Ftikas et al., 2010; Gorianovas et al., 2013; Liamopoulou et al., 2015; Ratel et al., 2015; Äyrämö et al., 2017; Birat et al., 2018; Lazaridis et al., 2018; Weinstein et al., 2018; Piponnier et al., 2019a; Bontemps et al., 2019; Piponnier et al., 2019b). Our results are in line with the current literature and statistically confirm that children have higher endurance capacities than adults (i.e., longer TTF) and are less fatigable (i.e., lower relative decrease in muscle performance at isotime). Physiological differences between children and adults may explain the aforementioned observations (Ratel et al., 2006a).

First, differences in muscle fiber types distribution between children and adults could explain part of the differences in fatigue resistance capacity during exercise. The type of muscle fibers composing a muscle influences its resistance to fatigue, with muscles mainly proportioned in type II fibers being less resistant (Barclay, 1996; Hamada et al., 2003). Although the evidences on age-related muscle typology are scarce so far and obtained on small sample sizes, it has been suggested that children have a higher proportion of slow-twitch type I fibers than adults, as reported by the ∼65–70% type I fibers proportion in the vastus lateralis in children versus ∼ 47–57% in adults (Lexell et al., 1992; Sjöström et al., 1992). This is associated with a greater muscle oxidative capacity, as demonstrated in the forearm flexor muscles by a higher rate of post-exercise recovery in phosphocreatine and a faster rate of aerobic ATP production (Ratel et al., 2008). Similar results were reported in the gastrocnemius muscle (Taylor et al., 1997). This could have a major influence on fatigue resistance especially during intermittent fatiguing exercise where the recovery in energy substrates plays a key role, i.e., children have a greater ability to replenish their phosphocreatine stores. Also, differences in mitochondrial function and density could favor the children to better liberate and capture oxygen and/or use it (McCormack et al., 2011; Ratel and Blazevich, 2017). Nine out of the 15 meta-analyzed studies that investigated TTF differences between children and adults used experimental designs with intermittent fatiguing protocols (Armatas et al., 2010; Murphy et al., 2014; Ratel et al., 2015; Piponnier et al., 2019a; Bontemps et al., 2019; Piponnier et al., 2019b; Woods et al., 2019; Piponnier et al., 2020; Woods et al., 2020). All of these nine studies reported longer TTF in children, with only three studies reporting the differences to be non-significant. Interestingly, five (Berthoin et al., 2003; Leclair et al., 2011; Patikas et al., 2013; Tanina et al., 2017; Bar-Yoseph et al., 2019) of the six studies that used continuous fatiguing protocols found no differences in exercise duration between children and adults (Table 2). This confirms that differences in metabolic profiles between children and adults could play a major role on the fatiguing resistance capacity especially when intermittent designs of exercise are used.

Second, the higher muscle mass involved during exercise in adults could account for the observed differences in muscle endurance and fatigability. Higher intramuscular pressure could occur during exercise in adults, thus increasing vascular occlusion and limiting metabolite removal and energy substrate replenishment (Ratel et al., 2008; Kappenstein et al., 2013), potentially leading to lower TTF. These peripheral alterations could interact with psychophysiological mechanisms that may play a fundamental role in determining the premature exercise ending in adults. A higher metabolic by-products accumulation, e.g., H^+^ accumulation (Blain et al., 2016), would increase the activation of metabosensitive group III/IV muscle afferents (Amann et al., 2015). These afferents project their input to various sites within the central nervous system (Craig, 1995; Craig, 2003). The central integration of afferent feedbacks [together with the increased corollary discharge, e.g., Amann et al. (2010)] may lead to increased sensations (e.g., effort, pain) involved in exercise termination (Kayser, 2003; Amann et al., 2011). One could speculate that the greater metabolic perturbations in adults would increase these sensations at a higher rate than in children thus leading to a premature exercise ending. These differences in perception of effort [usually reported in these studies into a single “Gestalt” perception, including other sensations like fatigue, discomfort as suggested in the seminal definitions of perceived exertion, e.g., Robertson et al. (1997); Borg (1998)] between children and adults have been addressed in a review (Ratel et al., 2006a), with a common observation that children rate their effort lower than adults (Bar-Or, 1977; Bar-Or, 1989; Ratel et al., 2004; Ratel et al., 2006b), supporting our previous assumption. However, this argument must be balanced with the fact that youth tend to score lower ratings of perceived effort during exercise (Huebner et al., 2014). In the latter study, the median maximum rating for leg exertion in children after cycling was only slightly greater than half the maximum possible value of 10 using the Borg CR10 scale (Borg, 1998). This observation has been confirmed by others (Bar-Or, 1977; Lamb, 1995; Barkley and Roemmich, 2008). This low-rating tendency could be due to the fact that children are unable to correctly understand the scale or properly gauge their perceived exertion, notably because of a lack of previous experiences (Huebner et al., 2014).

The differences in metabolic profile between children and adults could also give some clues to the observed differences in fatigability. The exercise duration has a direct influence on fatigability, with longer exercise inducing greater decrement muscle in performance (Millet, 2011). Then, it would have been incorrect to analyze fatigability data for experimental designs that used exercises of different duration. We thus performed isotime analysis to increase the robustness of our interpretation (Nicolò et al., 2019). For the same exercise duration, it is likely that children prevented the recruitment of high-threshold motor units, partly explaining the lower level of fatigability at isotime. This argument could be valuable for submaximal and maximal exercises if one considers that children have a greater activation deficit that adults. As reported in Table 3, a greater decrement in VA was observed in children after exercise, suggesting that children exhibited more central fatigue than adults. This may be in favor of a specific neural regulation in children during fatiguing exercises that could partly explain our results at isotime. In addition to the common argument of a lower capacity for spatial recruitment in children than in adults, i.e., lesser type-II motor-unit utilization (Dotan et al., 2012), one should also consider differences in temporal recruitment with differences in firing rates of the active motor units between children and adults. Direct evidences for this latter point are lacking so far, and the emergence of novel investigation technique (e.g., high-density electromyography) could help to obtain a more precise overview of differences in motor unit recruitment between children and adults. Last, the low activation level in children could allow the organization of motor units rotation (Ratel and Martin, 2015), which is much more difficult when the level of activation are high.

Further, because of their specific muscle phenotype, adults develop peripheral alterations at a higher rate when compared to children for the same relative exercise duration (see Figure 2 in the following references: Ratel et al. (2015); Bontemps et al. (2019); Piponnier et al. (2019a)) or at exercise termination (see the Pt-related data in Table 3), providing another possibility for the higher level of fatigability recorded at isotime in adults.

While the meta-analyses confirmed that children are 1) less fatigable and 2) able to sustain exercise at a given intensity longer than adults, one could speculate that the role of some specific physiological functioning, e.g., muscle metabolism, involved in the performance would vary as a function of the exercise modality, i.e., type of exercise (isometric versus dynamic) and exercise duration (short versus long-duration exercise), and that it could play a major role in the reported differences in TTF and fatigability between children and adults.

### 4.2 Differences in TTF and performance fatigability between children and adults depend on the modality of exercise

Sustained or intermittent isometric contractions at a single joint are common to evaluate TTF and fatigability. However, the conclusions derived from these contraction modalities do not necessarily apply for dynamic exercises where the physiological demands and the muscle mass involved in the exercise are different (Enoka and Stuart, 1992; Carroll et al., 2017). Considering the major changes in body size and physiological function over the course of growth and development (Cooper et al., 1987; Cooper et al., 2014), one may expect an influence of the type of exercise on the observed differences in TTF and fatigability between children and adults.

Among the 15 studies that looked at TTF differences between children and adults, 11 used an isometric modality (Armatas et al., 2010; Patikas et al., 2013; Hatzikotoulas et al., 2014; Ratel et al., 2015; Tanina et al., 2017; Piponnier et al., 2019a; Bontemps et al., 2019; Piponnier et al., 2019b; Woods et al., 2019; Piponnier et al., 2020; Woods et al., 2020) while four used a dynamic modality that included running and cycling exercises (Berthoin et al., 2003; Leclair et al., 2011; Bar-Yoseph et al., 2019) as well as concentric MVCs (Murphy et al., 2014). We found that children had longer TTF than adults when isometric exercises were performed with no differences for dynamic exercises. Eight out of the 11 isometric studies used intermittent fatiguing protocols. We can speculate that the use of intermittent exercises favored the children in sustaining the exercise for a longer duration, thanks to their higher muscle oxidative activity and their faster regulation of blood acid-base balance. Interestingly, two out of the three studies that used continuous isometric fatiguing protocols showed no differences in TTF between children and adults (Patikas et al., 2013; Tanina et al., 2017). The nature of the exercise (i.e., intermittent versus continuous) could explain the absence of differences between children and adults for the four studies that used dynamic exercises. Indeed, three out of these four studies used continuous exercises and showed either no changes (Leclair et al., 2011) or higher TTF in adults (Berthoin et al., 2003; Bar-Yoseph et al., 2019), while the one that used an intermittent design showed higher TTF in children (Murphy et al., 2014). Overall, these results support the idea that differences in exercise duration between children and adults are more detectable when intermittent exercises are used. One should note, however, the large imbalance in the number of studies included in the quantitative analyses that used isometric (*n* = 11) versus dynamic (n = 4) exercises which could have prevented detecting possible differences between children and adults for this latter exercise modality.

Among the 19 included studies in fatigability analyses, seven used isometric exercises (Halin et al., 2003; Hatzikotoulas et al., 2009; Ratel et al., 2015; Piponnier et al., 2019a; Bontemps et al., 2019; Piponnier et al., 2019b; Piponnier et al., 2020) and 12 used dynamic exercises that included running (Äyrämö et al., 2017), cycling (Hebestreit et al., 1993; Buchheit et al., 2010; Birat et al., 2018; Weinstein et al., 2018) and jumping (Marginson et al., 2005; Ftikas et al., 2010; Gorianovas et al., 2013; Liamopoulou et al., 2015; Lazaridis et al., 2018) efforts as well as repeated concentric MVCs (De Ste Croix et al., 2009; Dipla et al., 2009). The results revealed that children were less fatigable than adults when performing dynamic rather than isometric exercises. This finding is not consistent with TTF analyses which did not reveal any differences in TTF for dynamic exercises. While any attempt to give a physiological explanation for this result would remain hazardous, one could nevertheless suggest that the use of isotime comparison to compute fatigability in our meta-analysis is a candidate. Isotime data for most children were reported away from the end of the exercise. For instance, isotime comparisons in a study from our group (Piponnier et al., 2019b) were made at the 10th MVC which corresponded to the lowest number of contractions performed by one adult participant while the mean number of contractions was 40 ± 18 (range: 15–79) in children, suggesting that most children were still far from exercise termination. While these two outcomes (i.e., TTF and fatigability) are commonly used interchangeably to inform on exercise-related performance [e.g., Clark et al. (2005)], our results evidenced that they are not. TTF considers the time to exhaustion which rely on physiological mechanisms but also on psychological and motivational ones (Pageaux, 2016). Fatigability, as evaluated in this meta-analysis, informs on what happened on the early-middle phase of the exercise, far from exhaustion for most participants.

We also investigated whether differences in TTF were impacted by the exercise duration. Children are more likely to engage in very short bursts of intense physical activity interspersed with varying intervals of low to moderate intensity (Bailey et al., 1995). This is consistent with our results showing higher between-group differences, favoring children 1) for short compared to long-duration exercises and 2) for intermittent exercises. The classification of short versus long-exercise duration indirectly reflects the exercise intensity that was performed during the exercise. Six out of the seven studies classified in the short-duration category used high or maximal intensity of exercise (Berthoin et al., 2003; Hatzikotoulas et al., 2014; Murphy et al., 2014; Bontemps et al., 2019; Piponnier et al., 2019b; Piponnier et al., 2020) and five out of the seven studies classified in the long-duration category used moderate and submaximal intensities (Leclair et al., 2011; Patikas et al., 2013; Bar-Yoseph et al., 2019; Woods et al., 2019; Woods et al., 2020). Higher oxidative capacities in children could have favored a most effective recovery between intense exercise bouts explaining why they lasted longer than adults. Moreover, the perception of effort reported in children is often lower than in adults for short/intense bouts of exercise (i.e., up to 10 min) (Bar-Or and Rowland, 2004; Ratel et al., 2006a). This is likely due to differences in the way by which peripheral and central signals are integrated (Bar-Or and Rowland, 2004). Then, the perception of effort may increase at a higher rate in adults for short-duration and high-intensity exercises, contributing to earlier exercise termination, possibly explaining why children performed better for short-than long-duration exercises. Finally, differences in thermoregulation processes have been reported between children and adults (Notley et al. (2020) for a review). For instance, children present a lower rate of sweating than adults that is known to have a negative influence on body temperature regulation during prolonged exercise. This could lead to a higher metabolic demand relative to body mass in children, thus lowering the exercise economy (Rowland, 2008) and impacting exercise performance for long-duration exercises, especially involving whole-body tasks.

Overall, both the type of exercise and exercise duration can modulate the reported differences in fatigability and TTF between children and adults. Beyond these two modalities, it seems that the intermittent versus continuous design of exercise plays a significant role in these differences.

### 4.3 Physiological changes during maturation influence the exercise-related performance

Because significant changes occur in physiological systems during the transition from childhood to adulthood, there is a necessity to make a clear distinction between children and adolescents in the literature to have a precise overview of their potential differences in exercise-related performance especially when compared to adults. To apprehend the maturation-related influence on exercise performance in the best possible way, it would have been interesting to report children *versus* adolescents comparisons in dedicated meta-analysis. It was, however, not the primary purpose of this systematic review so as the inclusion criteria were not chosen in that way. Actually, only four and three studies could have been included in meta-analysis comparing children and adolescents for potential differences in fatigability and TTF, respectively, which is obviously too low to provide any robust and interpretable data. However, and when possible and relevant, children *versus* adolescents differences are descriptively discussed in the following paragraphs.

Differences in TTF and fatigability between adolescents and adults were investigated in only five (Pullinen et al., 2002; Pullinen et al., 2011; Tibana et al., 2012; Piponnier et al., 2019a; Bar-Yoseph et al., 2019) and seven (Kanehisa et al., 1995; Pullinen et al., 2002; Dipla et al., 2009; Buchheit et al., 2010; Willcocks et al., 2014; Äyrämö et al., 2017; Piponnier et al., 2019a) studies, respectively. Our analysis showed a longer but not significant (*p* = 0.09) TTF in adolescents together with a lower level of fatigability, when compared to adults. Only few studies compared children, adolescents and adults within the same experimental design. The results regarding TTF are controversial. Some authors reported longer TTF in children and adolescents when compared to adults, and a trend (*p* = 0.05) for longer TTF in children than adolescents (Piponnier et al., 2019a). Others reported longer TTF in adolescents and adults than children during running exercises (with no differences during cycling) (Bar-Yoseph et al., 2019). Eight studies investigated differences in fatigability between the three populations, some of them being not included in the statistical analyses because of methodological concerns (Ratel et al., 2002; Chen et al., 2014). Overall, adolescents are more fatigable than children (Ratel et al., 2002; Faigenbaum et al., 2008; Dipla et al., 2009; Chen et al., 2014; Piponnier et al., 2019a) but less than adults.

This confirms that growth and maturation influence the level of fatigability likely because of specific neuromuscular changes that are attributed to the puberty (Ratel and Martin, 2015). First, adolescents engage a higher muscle mass during the exercise (Van Praagh and Doré, 2002). This could be the origin of greater metabolic perturbations, especially because of greater intramuscular pressure during exercise. Differences in muscle typology [i.e., higher proportion of type II muscle fibers in adolescents, e.g., Glenmark et al. (1994)] or in energy metabolism [i.e., lower oxidative activity for ATP synthesis in adolescents, e.g., Berg and Keul (1988)] contribute to the higher level of fatigability observed in adolescents. Second, adaptations within the central nervous system during the maturation process could contribute to these differences. Children are less able to voluntarily recruit their motor units during exercise, likely due to an immaturity of the corticospinal pathway (Nezua et al., 1997). Besides this lower recruitment capacity, they would recruit a higher relative proportion of slow-twitch fibers and would benefit from a more organized and efficient motor unit rotation (Ratel and Martin, 2015). Because of the limited number of studies that investigated how peripheral and central modulations could differentially impact the exercise-related performance between children and adolescents, these arguments remain speculative. Only two studies investigated in the same experimental design the neural and peripheral functioning in response to exercise in children, adolescents and adults. Our group recently reported that Pt amplitude was reduced in adolescents after an intermittent isometric exercise while it was not in children (Piponnier et al., 2019a), suggesting that contractile properties and/or excitation-contraction coupling was preserved in children while altered in adolescents (Table 3). In contrast, VA decreased at a similar level after exercise in children and adolescents, while it remained unchanged in adults, suggesting that the greater central fatigue in children and adolescents likely account for their lower degree of peripheral alterations than adults. These isolated results strengthen the hypothesis of an evolution in the maturation of the central nervous system during growth, with the tolerance of the central nervous system to peripheral alterations increasing during puberty (Hamada et al., 2003). Besides this pioneer theory claiming that peripheral functioning is preserved by central regulation in children, one should also consider that the explanation could directly come from the muscle functioning, i.e., the fatigue-resistant muscles of the children do not develop a large amount of fatigue independently of any central influence. Another study investigated the peripheral and central factors of fatigue after a 50-s maximal run (Äyrämö et al., 2017) and confirmed the aforementioned observations. Of note, a large delay (i.e., 6–12 min) separated the end of the exercise and the beginning of neuromuscular testing in the latter study (Äyrämö et al., 2017). Considering the rapid recovery of neuromuscular function that occurs within the first 2-min after exercise (Froyd et al., 2013; Gruet et al., 2014; Mira et al., 2017), these latest results should be interpreted with caution.

### 4.4 Limitations

Some limitations pertaining to our analyses must be acknowledged. First, most outcomes displayed a moderate to high level of heterogeneity, likely due to differences in experimental procedures, e.g., type of exercise (isometric versus dynamic, long versus short duration, intermittent versus continuous), muscles involved (e.g., lower versus upper limbs) and population characteristics. These high levels of heterogeneity are, however, common in this kind of quantitative analyses, e.g., Kruger et al. (2018). A step towards a standardization of experimental protocols to evaluate TTF and fatigability in children, adolescents and adults could help in making the results of individual studies more homogeneous to establish more robust conclusions. Second, the overall quality of the studies included in the meta-analysis was low (mean quality score of 4.3 ± 1.2 stars out of a maximum of 9 stars) which could have led to a biased estimation of the between-group differences in TTF and fatigability. For instance, the overall score of the comparability domain was low (e.g., 0.9 ± 0.7 stars out of a maximum of 2 stars) meaning that physical activity status in some studies was not rigorously controlled. This is an important observation because the level of physical activity influences TTF and fatigability (Murphy and Smith, 2010; Buchowski et al., 2013) and varies during childhood (Guinhouya et al., 2013). Most of the included studies monitored the level of physical activity by the mean of self-report that involved questionnaires or brief interviews. These subjective measures can misjudge the absolute level of physical activity (Prince et al., 2008). Objective measures (e.g., accelerometers) can increase the precision of this important outcome. Only a minor proportion (∼12%) of the included studies used objective measures to capture the level of physical activity (Hebestreit et al., 1993; Buchheit et al., 2010; Weinstein et al., 2018; Bar-Yoseph et al., 2019), and one of them did not match the participants for similar level of physical activity (Buchheit et al., 2010). Third, a risk of publication bias was suggested for fatigability indicating that our analyses could overestimate fatigability differences between children and adults. Last, we excluded some studies because of the lack of information [e.g., absence of standard deviation (Gaul et al., 1995; Ratel et al., 2002), no access to the Pre-versus Post-exercise relative changes (Chen et al., 2014), no access to the contraction duration during intermittent exercises (Soares et al., 1996; Faigenbaum et al., 2008)], and their inclusion could have slightly influenced the results obtained in the meta-analyses.

## 5 Perspectives

Children are able to sustain an exercise longer and are less fatigable than adults. The selected exercise modality, i.e., the type and the duration of the exercise, in addition with fundamental differences in physiological functioning influences the reported differences between children and adults. While differences may also exist between children and adolescents, the low number of studies so far prevent a robust interpretation. Further studies using novel experimental techniques (e.g., transcranial magnetic stimulation, high-density electromyography) should be considered to gain novel insights into the interplay that could exist between peripheral and central mechanisms throughout maturation and that could explain the reported differences between children, adolescents and adults. Here are some practical recommendations that could increase the robustness of data interpretation when TTF and fatigability are evaluated in children, adolescents and adults:

1) Monitoring the level of physical activity by objective measures and pairing participants according to this outcome.
2) Performing isotime analysis when fatigability is assessed.
3) Recording both TTF and fatigability to obtain complementary information on exercise performance.
4) Combining various methods to assess the specific role of the central nervous system in children’ fatigability.
5) Combining different exercise modalities within the same study to capture the whole facet of the differences in TTF and fatigability.

## Data Availability

The original contributions presented in the study are included in the article/Supplementary Material, further inquiries can be directed to the corresponding author.
