# Supplementary material for: Differences in time to task failure and fatigability between children and young adults: A systematic review and meta-analysis
Source: Front Physiol. 2022 Oct 31;13:1026012. doi: 10.3389/fphys.2022.1026012 (PMC9661393; doi:10.3389/fphys.2022.1026012)
Supplement: Supplementary file 1 [file Table1.DOCX]

**Supplementary material 1** Search strategy used in Medline. The same strategy was used for the three databases, and 2486, 1385 and 597 articles were found with Medline (Pubmed), Cochrane Library and Sport Discus, respectively.

| **Search** | **Query** | **Results** |
| --- | --- | --- |
| **#4** | Search (#1 AND #2 AND #3) | **2,486** |
| **#3** | Search: ((((“Muscle Strength”) OR “Strength” OR “Force” OR “Isometric Force” OR “Maximal Voluntary Contraction” OR “MVC” OR “Power” OR “Velocity” OR “Isometric” OR “Concentric” OR “Eccentric” OR “Isokinetic” OR “Isotonic” OR "time to task failure" OR "time to exhaustion" OR "endurance time"))) | **1,132,400** |
| **#2** | Search: “neuromuscular fatigue” OR "fatigue" OR "Exercise-Induced Fatigue" OR "fatigability" OR "muscle fatigue" OR "cycling exercise" OR "running exercise" OR "aerobic exercise" OR "anaerobic exercise" OR "isometric exercise" OR "dynamic exercise" | **121,925** |
| **#1** | Search: "children" OR "child" OR “adolescent” OR “pubescent” OR “prepubescent” OR “prepubertal” OR “pubertal” OR “youth” OR “teen” | **3,723,213** |
