# Supplementary figures and images for: Differences in time to task failure and fatigability between children and young adults: A systematic review and meta-analysis
Source: Front Physiol. 2022 Oct 31;13:1026012. doi: 10.3389/fphys.2022.1026012 (PMC9661393; doi:10.3389/fphys.2022.1026012)

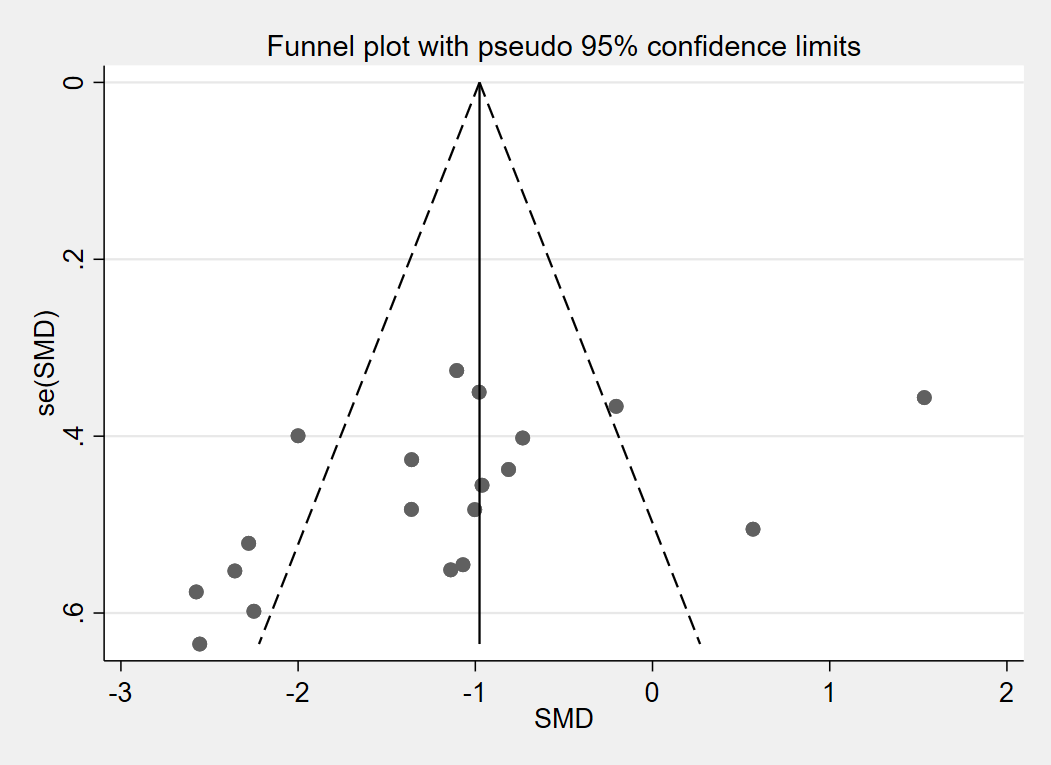

Supplement: Supplementary file 2 [file Image2.TIF]

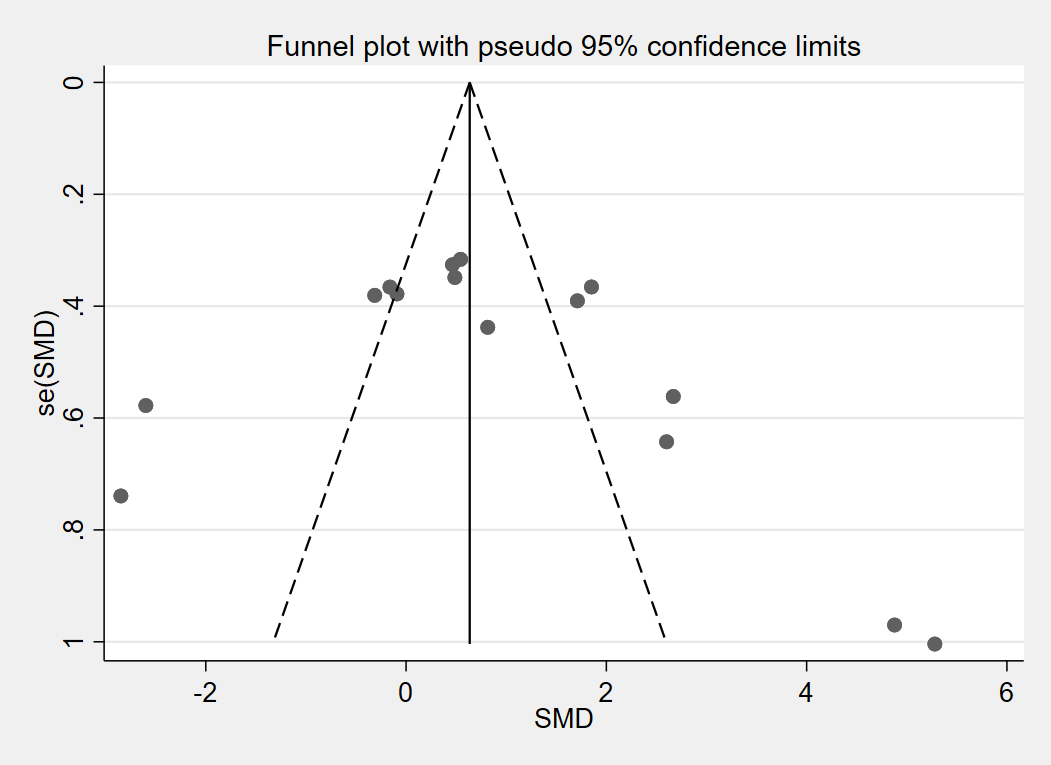

Supplement: Supplementary file 3 [file Image1.TIF]
