# Supplementary material for: Differences in time to task failure and fatigability between children and young adults: A systematic review and meta-analysis
Source: Front Physiol. 2022 Oct 31;13:1026012. doi: 10.3389/fphys.2022.1026012 (PMC9661393; doi:10.3389/fphys.2022.1026012)
Supplement: Supplementary file 4 [file Table2.DOCX]

**Supplemental material 2** Newcastle Ottawa Quality Assessment Scale (modified for cross-sectional studies)

***Selection: (Maximum 4 stars)***

1) Representativeness of the sample:

**a)** Representative of the average in the target populations (e.g. random sampling). 🢧🟊

**b)** Selected group of users. (e.g. local groups, university/school population) or no description of the sampling strategy. 🢧∅

2) Sample size:

**a)** Justified and calculated with appropriate methods. 🢧🟊

**b)** Not justified. 🢧∅

3) Ascertainment of participants’ health status.

**a)** Validated measurement tool to ensure subject’ health status (e.g. medical report, specific questionnaire). 🢧🟊🟊

**b)** Basic reporting of the health status (e.g. “healthy participants were recruited). 🢧🟊

**c)** No description of participants’ health status. 🢧∅

***Comparability: (Maximum 2 stars)***

1) Comparability of age groups based on controlling for confounding factors.

**a)** The study controls for physical activity status (e.g. specific physical activity questionnaire, accelerometry) and fitness level (e.g. maximal oxygen uptake) with a validated measurement tool (e.g. cardiopulmonary exercise testing with gas exchange measurement). 🢧🟊

**b)** The study controls for any additional factors (caffeine and alcohol consumption before the test and physical activity prior testing, any contraindication to physical activity). 🢧🟊

***Outcome: (Maximum 3 stars)***

1) Assessment of the main outcome:

**a)** Reliability of the fatiguing exercise in both children and adults (i.e. in the case of the authors reported the reliability of the fatiguing protocol using previous preliminary data or data published by other authors). 🢧🟊

**b)** Validated tool to measure time to task failure and/or performance fatigability (e.g. isometric/isokinetic ergometer, custom-built devices with force sensors). 🢧🟊

**c)** Non-validated tool or no description. 🢧∅

2) Statistical test:

**a)** The statistical test used to analyze the data is clearly described and appropriate, and the probability level (p-value) or effect size is reported. 🢧🟊

**b)** The statistical test is not appropriate, not described, or incomplete. 🢧∅
