# Supplementary material for: Differences in time to task failure and fatigability between children and young adults: A systematic review and meta-analysis
Source: Front Physiol. 2022 Oct 31;13:1026012. doi: 10.3389/fphys.2022.1026012 (PMC9661393; doi:10.3389/fphys.2022.1026012)
Supplement: Supplementary file 5 [file Table3.DOCX]

**Supplementary material 3** Quality assessment of all included studies.

|  | **Selection** | | |  | **Comparability** |  | **Outcome** | |  |  |
| --- | --- | --- | --- | --- | --- | --- | --- | --- | --- | --- |
| Study | Representativeness of the sample (🟊) | Sample size (🟊) | Health status (🟊🟊) |  | Comparability of age groups (🟊🟊) |  | Main outcome (🟊🟊) | Statistical test (🟊) |  | Total quality score (out of 9) |
| Armatas et al., (2010) | **–** | **–** | 🟊 | **1** | **–** | **0** | 🟊 | 🟊 | **2** | **3** |
| Äyrämö et al., (2017) | **–** | **–** | 🟊 | **1** | 🟊 | **1** | 🟊 | 🟊 | **2** | **4** |
| Bar-Yoseph et al., (2019) | **–** | **–** | 🟊🟊 | **2** | 🟊🟊 | **2** | 🟊🟊 | 🟊 | **3** | **7** |
| Berthoin et al., (2003) | **–** | **–** | 🟊 | **1** | 🟊 | **1** | 🟊 | 🟊 | **2** | **4** |
| Birat et al., (2018) | **–** | **–** | 🟊 | **1** | 🟊🟊 | **2** | 🟊🟊 | 🟊 | **3** | **6** |
| Bontemps et al., (2019) | **–** | **–** | 🟊🟊 | **2** | 🟊 | **1** | 🟊 | 🟊 | **2** | **5** |
| Buchheit et al., (2010) | **–** | 🟊 | 🟊 | **2** | 🟊 | **1** | 🟊 | 🟊 | **2** | **5** |
| De Ste Croix et al., (2009) | **–** | **–** | 🟊 | **1** | **–** | **0** | 🟊 | 🟊 | **2** | **3** |
| Dipla et al., (2009) | **–** | **–** | 🟊🟊 | **2** | 🟊 | **1** | 🟊🟊 | 🟊 | **3** | **6** |
| Ftikas et al., (2010) | **–** | **–** | 🟊 | **1** | **–** | **0** | 🟊 | 🟊 | **2** | **3** |
| Gorianovas et al., (2013) | **–** | **–** | 🟊 | **1** | **–** | **0** | 🟊 | 🟊 | **2** | **3** |
| Halin et al., (2003) | **–** | **–** | 🟊 | **1** | **–** | **0** | 🟊 | 🟊 | **2** | **3** |
| Hatzikotoulas et al., (2009) | **–** | **–** | 🟊 | **1** | **–** | **0** | 🟊 | 🟊 | **2** | **3** |
| Hatzikotoulas et al., (2014) | **–** | **–** | 🟊 | **1** | 🟊 | **1** | 🟊 | 🟊 | **2** | **4** |
| Hebestreit et al., (1993) | **–** | **–** | 🟊 | **1** | 🟊🟊 | **2** | 🟊🟊 | 🟊 | **3** | **6** |
| Kanehisa et al., (1995) | **–** | **–** | 🟊 | **1** | **–** | **0** | 🟊 | 🟊 | **2** | **3** |
| Lazaridis et al., (2018) | **–** | **–** | 🟊 | **1** | 🟊 | **1** | 🟊 | 🟊 | **2** | **4** |
| Leclair et al., (2011) | **–** | **–** | 🟊 | **1** | 🟊 | **1** | 🟊 | 🟊 | **2** | **4** |
| Paraschos et al., (2015) | **–** | **–** | **–** | **0** | **–** | **0** | 🟊 | 🟊 | **2** | **2** |
| Marginson et al., (2005) | **–** | **–** | 🟊🟊 | **2** | 🟊 | **1** | 🟊 | 🟊 | **2** | **5** |
| Murphy et al., (2014) | **–** | **–** | 🟊🟊 | **2** | 🟊 | **1** | 🟊 | 🟊 | **2** | **5** |
| Patikas et al., (2013) | **–** | **–** | 🟊 | **1** | 🟊 | **1** | 🟊 | 🟊 | **2** | **4** |
| Piponnier et al., (2019b) | **–** | **–** | 🟊 | **1** | 🟊 | **1** | 🟊 | 🟊 | **2** | **4** |
| Piponnier et al., (2019a) | **–** | **–** | 🟊 | **1** | 🟊 | **1** | 🟊 | 🟊 | **2** | **4** |
| Piponnier et al. ,(2020) | **–** | **–** | 🟊 | **1** | 🟊 | **1** | 🟊 | 🟊 | **2** | **4** |
| Pullinen et al., (2002) | **–** | **–** | 🟊 | **1** | 🟊 | **1** | 🟊 | 🟊 | **2** | **4** |
| Pullinen et al., (2011) | **–** | **–** | 🟊 | **1** | 🟊 | **1** | 🟊 | 🟊 | **2** | **4** |
| Ratel et al., (2015) | **–** | **–** | 🟊🟊 | **2** | 🟊 | **1** | 🟊 | 🟊 | **2** | **5** |
| Tanina et al., (2017) | **–** | **–** | 🟊 | **1** | 🟊 | **1** | 🟊 | 🟊 | **2** | **4** |
| Tibana et al., (2012) | **–** | **–** | 🟊🟊 | **2** | 🟊 | **1** | 🟊 | 🟊 | **2** | **5** |
| Weinstein et al., (2018) | **–** | **–** | 🟊🟊 | **2** | 🟊🟊 | **2** | 🟊🟊 | 🟊 | **3** | **7** |
| Willcoks et al., (2014) | **–** | **–** | 🟊 | **1** | **–** | **0** | 🟊 | 🟊 | **2** | **3** |
| Woods et al., (2019) | **–** | **–** | 🟊 | **1** | 🟊🟊 | **2** | **–** | 🟊 | **1** | **4** |
| Woods et al., (2020) | **–** | **–** | 🟊 | **1** | 🟊🟊 | **2** | **–** | 🟊 | **1** | **4** |

| **Rating frequency for each domain considering the McPheeters classification- *Number of study (total percentage)*** | | | |
| --- | --- | --- | --- |
|  | Selection | Comparability | Outcome |
| Good | 0 (0) | 6 (18) | 5 (15) |
| Fair | 9 (26) | 19 (56) | 27 (79) |
| Poor | 25 (74) | 9 (26) | 2 (6) |
